# Enhancing the light olefin selectivity of an iron-based Fischer–Tropsch synthesis catalyst by modification with CTAB†

**DOI:** 10.1039/c8ra04622k

**Published:** 2018-09-14

**Authors:** Chuanxue Zhu, Yingxin Liu, Chao Huo, Huazhang Liu

**Affiliations:** Institute of Industrial Catalysis, Zhejiang University of Technology Hangzhou 310032 Zhejiang PR China zhucx@zjut.edu.cn chaohc@zjut.edu.cn hzliu@zjut.edu.cn +86-571-88320815 +86-571-88320815; Research and Development Base of Catalytic Hydrogenation, College of Pharmaceutical Science, Zhejiang Technology Hang-zhou 310032 Zhejiang PR China yxliu@zjut.edu.cn

## Abstract

The effects of the surfactant hexadecyltrimethylammonium bromide (CTAB) on the catalytic performance of a manganese-promoted iron (FeMn) catalyst for the Fischer–Tropsch to olefin (FTO) reaction were investigated. The use of the CTAB-assisted FeMn catalyst resulted in the production of light olefin (C_2–4_^=^) selectivity of up to 55.45% with a ratio of olefin to paraffin among the C_2_–C_4_ hydrocarbons as high as 7.75 under industrially relevant conditions (320 °C, 1.0 MPa, H_2_/CO ratio of 1.5 (v/v), GHSV = 4200 h^−1^). The characterization results indicate that CTAB has a great influence on the structure, composition, chemical state, and catalytic performance of the iron-based catalyst. Most interestingly, a greater amount of Mn promoter was found to be dispersed on the surface of α-Fe_2_O_3_, rather than being dissolved into the α-Fe_2_O_3_ lattice when CTAB was employed, which contributed towards enhancing the promotional effects of the Mn promoter, leading to the formation of certain surface-specific activity sites.

## Abstract 2

The effects of the surfactant hexadecyltrimethylammonium bromide (CTAB) on the catalytic performance of a manganese-promoted iron (FeMn) catalyst for the Fischer–Tropsch to olefin (FTO) reaction were investigated.

## Graphical abstract

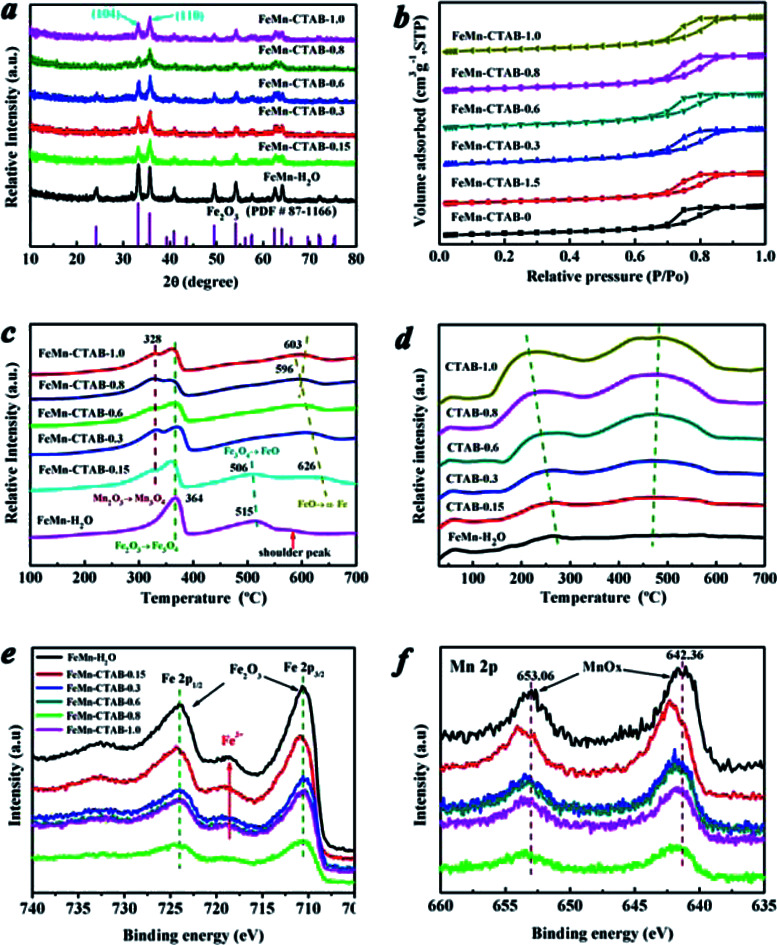

## 1. Introduction

Light olefins (C_2–4_^=^), which include ethylene, propylene and butylenes, are widely used in the chemical industry as key building blocks.^1,2^ Traditionally, light olefins are produced by naphtha cracking or fluid catalytic cracking, raising many concerns about the availability of petroleum-based feedstocks and their serious impact on the environment.^3,4^ Therefore, alternative routes for their production have been explored. For example, light olefins can be produced *via* direct routes, namely, the oxide–zeolite (OX–ZEO) process and Fischer–Tropsch to olefins (FTO) reaction.^2,3,5–7^ In both of these processes, synthesis gas, a mixture of CO and H_2_, which can be derived from coal, natural gas, and renewable biomass, is employed as a feedstock.^8^ The FTO reaction has received renewed interest in recent decades because of its simplified operation conditions and low energy consumption.^4,9^ However, the FTO reaction is based on the surface polymerization of CH_*x*_ species to form hydrocarbons, and the selectivity of C_2_–C_4_ hydrocarbon products is limited by the so-called Anderson–Schulz–Flory (ASF) distribution, which is no more than 58%. Furthermore, high methane selectivity is a big challenge for the FTO process.^4,5,10^ Other negative factors, such as carbon deposition and catalyst stability, remain severe problems for the FTO reaction. Generally, iron-based and cobalt-based catalysts are expected to be used for highly efficient FTO reactions.^11,12^ Iron-based catalysts are commonly applied in the FTO reaction, due to them being favorable for light olefin production, low cost and having a high activity for the water–gas shift reaction (WGS).^11,13^

Fischer–Tropsch chemistry is very complex and subtle, and can be greatly influenced by reaction conditions and catalyst properties.^14–16^ Recently, various iron-based catalysts have been designed for improving light olefin selectivity by proper catalyst modification, such as optimizing the catalyst composition and structure.^17–21^ The modification of iron-based FTO catalysts with promoters has been shown to be an effective way of tuning the selectivity of products.^20,22,23^ As an electron promoter, manganese can enhance the selectivity of olefins by suppressing methane selectivity and the α-olefin secondary reaction.^19^ Manganese has specific effects on iron-based catalyst properties, including changing the surface basicity and the electronic state of the active phase, and effecting the dispersion of iron, which consequently affect the catalyst reduction and carbonization activities.^19,23–25^ Recently, the modulation of surfactant has been reported to exert a great influence on nucleus formation and crystal growth during co-precipitation by adsorbing on the formed particle surfaces, which can efficiently protect the particles from agglomeration and even tailor the surface properties of the catalyst.^5,26–28^

Iron carbides have been reported to be the catalytic active phase of iron-based catalysts during the Fischer–Tropsch synthesis (FTS) reaction in many studies.^29–31^ Suitable iron carbide particle size or shell thickness was found to be essential for high catalytic activity.^14,32^ However, the exact role that different carbides play in syngas conversion, *via* the FTS or FTO reactions, is controversial. This is why the field of FTS has received wide attention. As reported, the use of χ-Fe_5_C_2_ (Hägg carbide) favours the production of C_5+_ hydrocarbons and oxygenates,^29,33^ while θ-FeC_3_ (iron carbide) results in the formation of light olefins.^19^ Even so, the understanding of iron carbide species in the FTO reaction has met with limited success.

In our work, the catalysts were prepared using a surfactant hexadecyltrimethyl ammonium bromide (CTAB)-associated metal salt solution precursor *via* a co-precipitation route. The addition of CTAB led to effective control of the size and shape of the formed metal oxide nanoparticles,^34–39^ which had a great influence on the nature of the catalyst surface and its performance. A narrow particle-size distribution with smaller particles was obtained when sufficient surfactant (CTAB) was employed, which did well in inhibiting carbon deposition during the FTO reaction. Moreover, a larger amount of Mn promoter was found at the surface of α-Fe_2_O_3_, rather than being dissolved into the α-Fe_2_O_3_ lattice, resulting in a low interaction between Fe–Mn, which contributed to the promotional effects of Mn on the iron-based FTO catalyst.^19,40^ Here, the effects of CTAB on the catalyst properties, involving the structure, surface chemical state and catalytic performance were investigated in detail using several characterization methods.

## 2. Experimental section

### 2.1 Synthesis of the catalysts

The FeMn catalysts were prepared *via* a modified co-precipitation method. Typically, an appropriate amount of iron(iii) nitrate nonahydrate (Fe(NO_3_)_3_·9H_2_O) and manganese(ii) nitrate tetrahydrate (Mn(NO_3_)_2_·4H_2_O) were dissolved into deionized water to form a 0.136 M metal nitrate solution (the nominal molar ratio of Mn/Fe was 0.08). This salt solution was introduced into a solution of CTAB (350 mL) to obtain a mixed solution, which was preheated to 60 °C. Then, a solution of NH_3_·H_2_O (1.0 M, 300 mL) was slowly dropped into the above mixed solution in a beaker (pH ∼ 9.5) and was mechanically stirred (400 rpm) for 1.5 h. A constant temperature of 60 ± 0.5 °C was maintained during the precipitation process. After aging for 12 h at room temperature, the obtained suspension was filtered, washed thoroughly with deionized water, dried overnight at 120 °C and then calcined at 400 °C for 5 h under static air. The obtained catalysts were denoted as FeMn–CTAB-*γ*, in which *γ* is the molar ratio of CTAB to Fe atoms. For comparison, the catalyst was prepared without CTAB following a similar procedure, and was named as FeMn–H_2_O.

### 2.2 Catalyst characterization

The texture properties of the samples were measured by nitrogen physisorption at −196 °C in ASAP 2010 (Micromeritics, USA). Prior to the adsorption measurements, the samples were evacuated under vacuum at 200 °C for 6 h. The specific surface area was calculated using the Brunauer–Emmett–Teller (BET) equation, and the total pore volume and average pore size were determined using the Barrett–Joyner–Halenda (BJH) method.

The structures of the catalysts were determined using an X-ray diffractometer (XRD) (Rigaku, Thermo ARL SCINTAGX TRA) equipped with CuKα radiation (*λ* = 1.54050 Å, 40 kV, 40 mA) in the 2*θ* angle range from 10–80° at a scanning speed of 4° min^−1^. The *in situ* XRD patterns of the catalyst were recorded under a flow of H_2_ (30 mL min^−1^) during the stepwise heating of the sample from 25 to 500 °C at a rate of 5 °C min^−1^.

The actual compositions of the samples were analyzed using X-ray fluorescence (XRF, Rigaku ZSX Primus II) and energy-dispersive spectrometers (EDS, Thermo NORAN, USA) attached to a scanning electron microscope. The near surface chemical information of the materials was analyzed by X-ray photoelectron spectroscopy (XPS, Kratos AXIS Ultra DLD, Japan) using an Al Kα X-ray source, where the base pressure of the chamber was less than 2 × 10^−8^ Pa. The binding energies (BEs) were calibrated relative to adventitious carbon using the C 1s peak at 284.8 eV.

The morphology of the catalysts was characterized by transmission electron microscopy (TEM) on a Hitachi H-600 electron microscope operated at an accelerating voltage of 100 kV. The TEM specimens were prepared by ultrasonically suspending a powder sample in ethanol, and then drops of the suspension were deposited on a carbon-coated copper grid and dried at room temperature before analysis.

The reduction behavior was measured by hydrogen temperature-programmed reduction (H_2_-TPR) experiments carried out in an ASAP 2020 (Micromeritics, America) using 5 vol% H_2_/95 vol% Ar (flow rate 50 mL min^−1^) as the reducing agent. About 30 mg of sample was pretreated under an Ar flow (20 mL min^−1^) at 200 °C for 2 h, and for the reduction, the sample was heated from room temperature to 700 °C at a rate of 10 °C min^−1^. The TPR profiles were recorded using the response of the thermal conductivity detector (TCD) of the effluent gas.

The basicity of the samples was determined by stepwise temperature-programmed desorption of carbon dioxide using the same equipment as that employed for the H_2_-TPR measurements. About 50 mg of catalyst was loaded into the reactor and was heated under a flow of He (50 mL min^−1^) from room temperature to 700 °C. The samples were subsequently cooled to 50 °C under the He flow, and then exposed to CO_2_ for 1 h, followed by purging with He for 1 h to remove the weakly adsorbed species. After this step, the temperature was increased to 700 °C at a rate of 10 °C min^−1^.

### 2.3 Catalyst evaluation

The FTS reaction was performed in a fixed-bed reactor with an inner tube diameter of 10 mm. Generally, the catalyst (60–100 mesh, 1.0 mL) was mixed with quartz sand (60–100 mesh, 5.0 mL) and then loaded into a stainless-steel reactor (the catalyst was diluted with the same size quartz sand to remove any temperature gradient within the catalyst bed). The gas flow was controlled by mass flow controllers (MFC). The reaction products passed over a 160 °C hot trap and a −1 °C cooling trap under working pressure, resulting in the collection of aqueous, liquid oil, and solid wax products. The residual outlet gas was analyzed online by gas chromatography (GC). The catalyst was reduced prior to the reaction in H_2_ at 400 °C under 1.0 MPa for 12 h with a gas hourly space velocity (GHSV) of 1500 h^−1^ (1500 mL h^−1^ mL_cat_^−1^). After that, the reactor was cooled down to 200 °C. Subsequently, the feed flow was switched to synthesis gas (volume ratio H_2_/CO = 1.5), with the pressure maintained at 1.0 MPa, and the reactor was heated to 320 °C at 4 °C min^−1^ with a GHSV of 4200 h^−1^. The gaseous reaction products were analyzed online using a gas chromatograph (SHIMADZU GC 2014ATF) equipped with two columns and two detectors. The analysis of the H_2_, CO, CO_2_, and CH_4_ content of the outlet gases was performed using a carbon molecular sieve column (TDX-1) with a thermal conductivity detector (TCD), using He as the carrier gas. The hydrocarbon (C_1_–C_5_) products were analyzed using a KCl modified alumina capillary column (19095P-K25) with an argon carrier and a hydrogen flame ionization detector (FID). The selectivity was calculated as the percentage of equivalent carbons present in the hydrocarbon product (C%).

The CO conversion was calculated on a carbon-atom basis, as follows:1
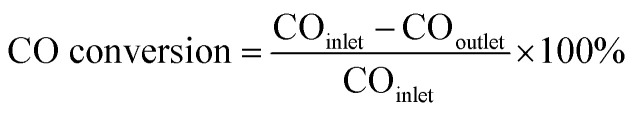
where CO_inlet_ and CO_outlet_ represent the moles of CO at the inlet and outlet, respectively.

The CO_2_ selectivity was calculated according to the equation:2
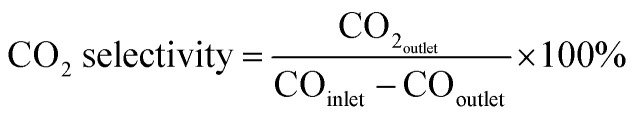
where CO_outlet_ represents the moles of CO_2_ at the outlet.

The selectivity of the individual product C_*n*_H_*m*_ was obtained from the equation:3
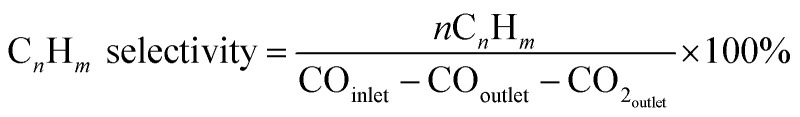
where C_*n*_H_*m* outlet_ represents the moles of C of the product at the outlet.

## 3. Results and discussion

**Fig. 1:**
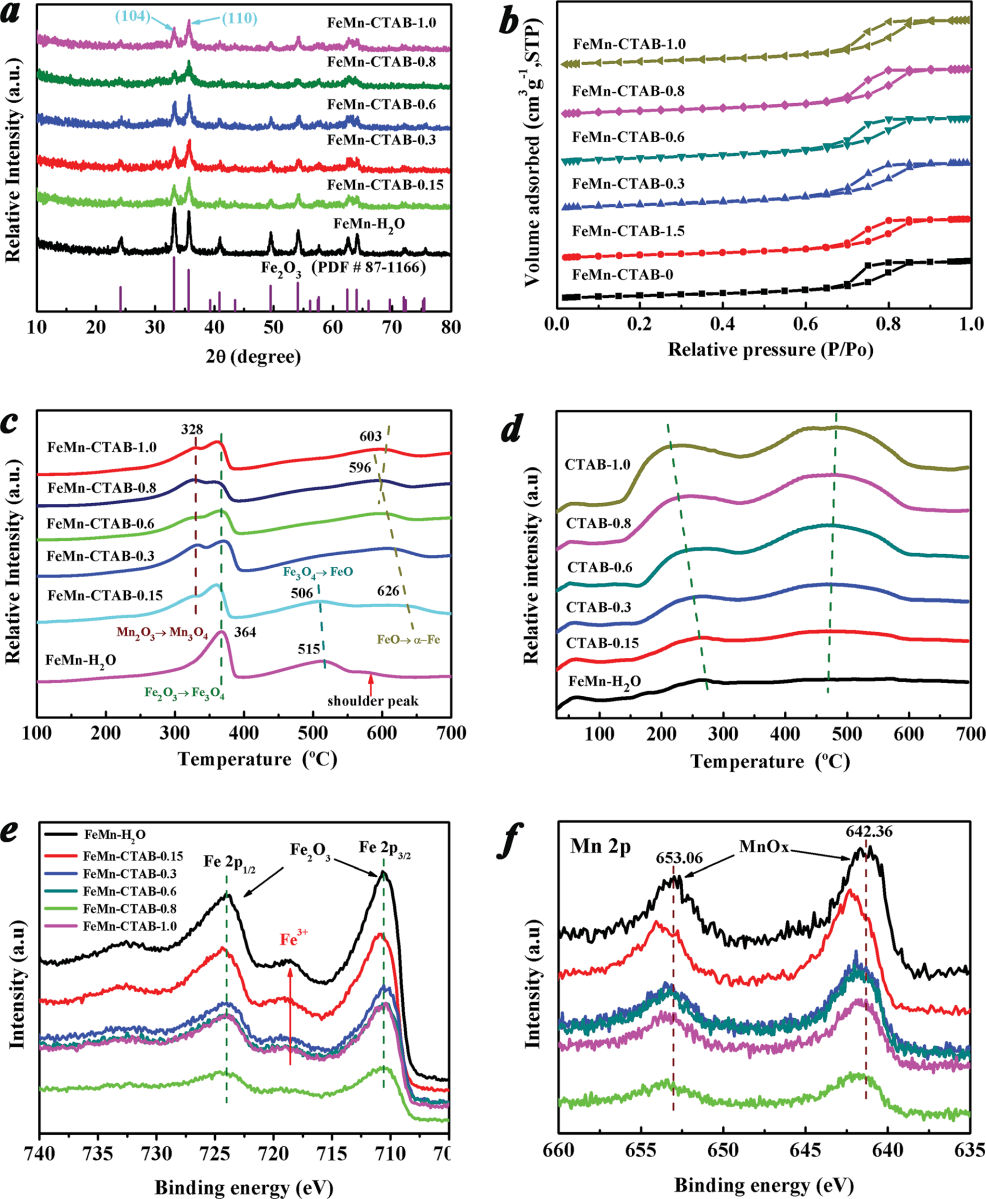
(a) XRD patterns, (b) N_2_ adsorption–desorption isotherms, (c) H_2_-TPR profiles, (d) CO_2_-TPD profiles, (e) Fe 2p XPS spectrum, (f) Mn 2p XPS spectrum of the calcined FeMn catalysts with different amounts of CTAB.

The XRD patterns of the calcined FeMn catalysts exhibit similar peaks at 2*θ* values of 24.2, 33.2, 35.6, 40.9, 49.5, 54.1, 62.4, and 64.0° (Fig. 1a), which can be assigned to the (012), (104), (110), (113), (024), (116), (214), (300) planes of α-Fe_2_O_3_ (hematite phase, PDF#87-1166), respectively. In addition, no MnO_*x*_ can be observed in all of the XRD patterns. However, the elemental analysis results showed that the Mn/Fe ratios on these sample surfaces are in the range of 0.12–0.17 (Table 1). This suggests that MnO_*x*_ was highly dispersed on the surface of the catalysts,^41^ or that the size of the crystalline MnO_*x*_ is out of the XRD detection limit.^42^ The broad peaks of the FeMn–CTAB catalysts indicate a very small crystallite size.^32^ Calculated *via* the Scherrer equation based on the (104) plane, the average α-Fe_2_O_3_ nanoparticle size was found to significantly decrease from 18.1 nm for FeMn–H_2_O to 6.1 nm for FeMn–CTAB-0.8 (Table S1†). These results suggest that reducing the nanoparticle size of the FeMn mixed oxide using a CTAB-associated iron and manganese oxide co-precipitation method, by suppressing particle aggregation in the preparation process, is an effective method.^34,35^ Furthermore, compared with that of the FeMn–H_2_O catalyst, there was a greater increase in the intensity of the peak of the (110) plane than that of the (104) plane upon the addition of CTAB, which is anomalous to the standard stick pattern (JCPDS card no. 033-0664). This result implies that the addition of CTAB in the preparation of iron-based catalysts dramatically influences the growth orientation of the α-Fe_2_O_3_ nanocrystals, resulting in the preferential exposure of the (110) plane.

**Table 1:** Catalytic performance of the FeMn catalysts with different amounts of added CTAB^a^

Catalysts	CO conv. (%)	CO_2_ sel. (%)	Hydrocarbon selectivity (C mol%, CO_2_-free)				O/P^b^
			CH_4_	C_2–4_ olefins	C_2–4_ paraffins	C_5+_ oxygenates^c^	
FeMn–H_2_O	91.31	41.04	15.29	30.16	13.75	40.80	2.19
FeMn–CTAB-0.15	88.30	36.99	13.59	29.81	10.78	45.82	2.77
FeMn–CTAB-0.3	73.57	29.65	11.38	35.25	9.08	44.29	3.88
FeMn–CTAB-0.6	55.41	23.62	14.69	44.13	9.18	32.0	4.81
FeMn–CTAB-0.8	34.76	14.0	18.04	55.45	7.16	19.36	7.75
FeMn–CTAB-1.0	63.70	21.13	12.31	37.72	6.67	43.30	5.66

a Catalysts were *in situ* reduced at 400 °C for 12 h and tested at 320 °C, 1.0 MPa, GHSV = 4200 h^−1^, H_2_/CO = 1.5 (v/v), at TOS = 20 h.

b O/P represents the molar ratio of olefin to paraffin in the C_2_–C_4_ range of hydrocarbons.

c C_5+_ are the hydrocarbons that were analyzed online by gas chromatography (GC); oxygenates are from the 160 °C hot trap and −1 °C cooling trap.

The N_2_ adsorption–desorption isotherms (Fig. 1b) show that all of the samples exhibit type IV isotherms with clear H_2_(b) hysteresis loops due to capillary condensation in the mesoporous materials.^32^ Two peaks can be observed from the pore size distribution curve of the FeMn–H_2_O catalyst (Fig. S1†), suggesting the co-existence of two types of pores. The small pores centered at around 5–12 nm are generated from the intra-aggregation of Fe_2_O_3_ and the large pores observed at around 80 nm are caused by inter-aggregation of the catalyst.^32,43^ It is noteworthy that the size of the intra-aggregated pores slightly shifts towards a smaller pore size, while the inter-aggregated pores become broader and then disappear upon the addition of CTAB, indicating that the aggregated particle size significantly decreased and the aggregation of the catalysts was effectively suppressed.^43^

The BET surface areas and total pore volumes of the FeMn catalysts increased significantly from 73.22 m^2^ g^−1^ and 0.23 m^3^ g^−1^ for the FeMn–H_2_O catalyst to 135.65 m^2^ g^−1^ and 0.30 m^3^ g^−1^ for the FeMn–CTAB-0.8 catalyst (Table S1†). The molar ratios of Mn/Fe on the surfaces of the catalysts were measured by XPS and were found to be much higher than that of the bulk determined by XRF, which suggests that Mn was mainly dispersed on the surface of the α-Fe_2_O_3_ particles (Table S1†). No residual elemental bromine (Br) was detected, which indicates that the Br species was completely removed during decomposition of CTAB by calcination (400 °C, 5 h). The TGA-MS result in Fig. S2† proves that the surfactant was entirely evaporated and that no residual carbon source from the CTAB was present in the catalyst, as observed by a lack of signal for CO/CO_2_.

The redox properties of the catalysts are shown in Fig. 1c. The reduction peaks in the range of 280–390 °C, 390–550 °C and 550–630 °C in the H_2_-TPR profile of the FeMn–H_2_O catalyst were ascribed to the processes of α-Fe_2_O_3_ to Fe_3_O_4_, Fe_3_O_4_ to FeO and FeO to metallic Fe,^23,32,41,42^ respectively. The first reduction peak gradually divided into two distinct peaks upon the addition of CTAB. The peak in the range of 280 to 345 °C corresponded to the reduction of α-Mn_2_O_3_ to Mn_3_O_4_,^23,41,42^ of which the peak area gradually enhanced, as presented in Table S2,† suggesting that there was a larger amount of α-Mn_2_O_3_ exposed on the surface. The other peak in the range of 345 to 390 °C was assigned to the reduction of α-Fe_2_O_3_ to Fe_3_O_4_,^23,32^ however, the peak area showed the opposite trend to that of the previous peak (Table S2†), which might be due to the higher coverage of Mn promoter on the surface iron atoms, limiting the contact between the active Fe phase and H_2_.^19,40^

The intensity of the second peak of FeMn–H_2_O decreased when a low amount of CTAB was employed, and disappeared upon the further addition of CTAB, which indicates that the Fe_3_O_4_ was directly reduced to metallic Fe, and that FeO was not formed as an intermediate because it was unstable.^23,41^ It is possible that the increase in the amount of CTAB added inhibited the incorporation of Mn^2+^ ions into the FeO lattice, leading to a significant decrease in the interaction between Fe–Mn and a large amount of MnO_*x*_ species dispersing on the α-Fe_2_O_3_ surface.^23,24,42^ From the H_2_-TPR profiles, it was revealed that the third reduction peak intensity remarkably increased with peak temperature dropping to the lowest 596 °C for FeMn–CTAB-0.8 catalyst, caused by the higher dispersion of the metal oxides, smaller nanoparticles size, and larger BET areas were obtained upon addition of CTAB.^24,41^ The reduction process of the calcined FeMn–CTAB-0.8 catalyst in H_2_ was also studied by *in situ* XRD, as exhibited in Fig. S3,† which further revealed the reduction of α-Fe_2_O_3_*via* the processes of α-Fe_2_O_3_ to Fe_3_O_4_, Fe_3_O_4_ to FeO and FeO to metallic Fe.

The catalyst surface basicity was investigated by CO_2_-TPD, as shown in Fig. 1d. The first peak at a low temperature of around 60 °C in the CO_2_-TPD profile can be attributed to the desorption of CO_2_ weakly adsorbed in the bulk α-Fe_2_O_3_ phase.^24,42^ The other two peaks in the range of 130 to 330 °C and 330 to 660 °C can be attributed to the desorption of CO_2_ that interacted moderately with the surface medium-strong basic sites and the strongly chemisorbed CO_2_ that had some bearing on the surface strong basic sites,^24,42^ respectively. The peak intensities in the high temperature area of the CO_2_-TPD profile increased significantly upon the addition of CTAB (Table S3†), which indicates that CTAB remarkably enriched the basic sites on the catalyst surface. This may be due to the surface imperfections on the small particles as well as a greater amount of MnO_*x*_ species exposed to the surface, enhancing the number of basic sites.^43,44^ Moreover, the peak position shifted to a higher temperature, which suggests an increase in basicity.^42^

The Fe 2p XPS spectra of the catalysts are shown in Fig. 1e. The peaks positioned at around 710.28 and 724.08 eV (Table S4†) correspond to the Fe 2p_3/2_ and Fe 2p_1/2_ levels of Fe^3+^, respectively, together with Fe^3+^ satellite peaks detected at around 718.78 eV, verifying the formation of the α-Fe_2_O_3_ phase for all of the samples.^32,40^ Notably, there was a decrease in the Fe 2p_3/2_ peak intensity upon an increase in the amount of CTAB employed, implying there was a decrease in the number of Fe atoms on the surface.^9,40^ More noteworthy is that the Fe 2p_3/2_ peak position shifted to a lower binding energy upon an increase in the CTAB to Fe atom molar ratio in the preparation, indicating a higher electron density of surface Fe atoms (Fig. 1e).^9,19,40^

Fig. 1f reveals that Mn promoter is located on the surface of the catalysts. The Mn 2p XPS peak positions imply that various oxidized manganese species form upon the addition of CTAB, such as Mn^3+^ and Mn^4+^ for the promoted catalysts, as shown in Fig. S5,† which suggests that electron transfer occurs between the surface Mn and Fe atoms.^9^ It is worth pointing out that the molar ratio of Mn/Fe at the surface of the FeMn–CTAB-0.8 sample slightly decreased even if a greater number of Mn atoms were exposed on the surface, owing to the lower and higher number of particles of both Fe and Mn oxides. Furthermore, regardless of whether the Mn promoter was located on the α-Fe_2_O_3_ surface, or dissolved into the α-Fe_2_O_3_ lattice, it did not result in a significant change in the Mn/Fe ratio on the catalyst surface.

**Fig. 2:**
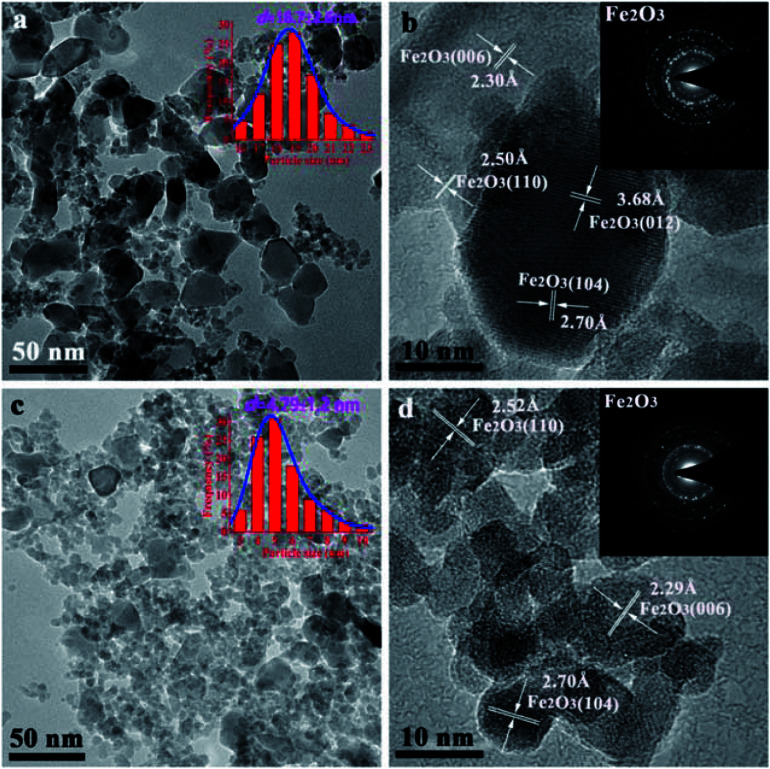
TEM images of the calcined FeMn–H_2_O and FeMn–CTAB-0.8 as-prepared catalysts, where *d* refers to the average particle size.

The morphologies of the calcined FeMn–H_2_O and FeMn–CTAB-0.8 catalysts were measured by TEM, as shown in Fig. 5. A much smaller nanoparticle size was observed for the samples with CTAB. The average particle size of the FeMn–H_2_O catalyst was 18.7 nm, estimated by randomly selecting 200–300 particles in the range of 10 to 35 nm (Fig. 2a), while for the FeMn–CTAB-0.8 catalyst, the nanoparticles were found to be in the range of 3–10 nm, except for a few particles with a larger size of around 19.0 nm. The average size of the nanoparticles was 4.79 nm, which can be observed in the particle size distribution (PSD) in Fig. 2c. Combining the information from the TEM images and the corresponding PSD (Fig. S4†), it can be considered that the addition of CTAB effectively decreases the nanoparticle size.^34–38^ Moreover, Fig. S4d† shows that a narrower particle-size distribution was obtained when sufficient CTAB was employed. The reason for this might be that CTAB effectively prevents the particles from agglomerating during the co-precipitation process.^34–38,45^ HR-TEM analyses demonstrated that such nanoparticles are composed of α-Fe_2_O_3_, with specifically exposed interplanar spacing facets of (006) (2.29 Å), (110) (2.52 Å), and (104) (2.70 Å) (Fig. 2d). No MnO_*x*_ was detected in the samples. These are results were found to be in good agreement with the XRD results. The selected area electron diffraction (SAED) pattern of FeMn–H_2_O revealed it to be polycrystalline, while that of the FeMn–CTAB-0.8 catalyst showed its single crystalline nature.

**Fig. 3:**
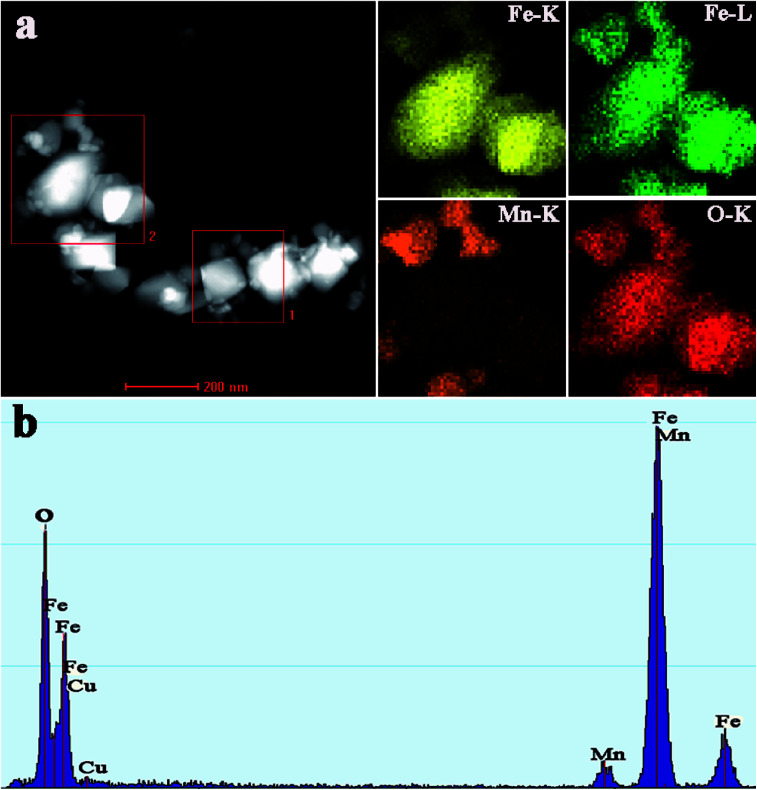
TEM mapping of the calcined FeMn–CTAB-0.8 catalyst.

EDS elemental mapping analyses were further employed to gain an insight into the actual distributions of Fe and Mn in the calcined FeMn–CTAB sample. As shown in Fig. 3a, it was found that the Fe patch corresponds to big nanoparticles, while the Mn patch was detected in the surrounding small nanoparticles and the O patch almost exhibits the same shape. These results strongly indicate that the Mn species is located on the surface of Fe_2_O_3_.

**Fig. 4:**
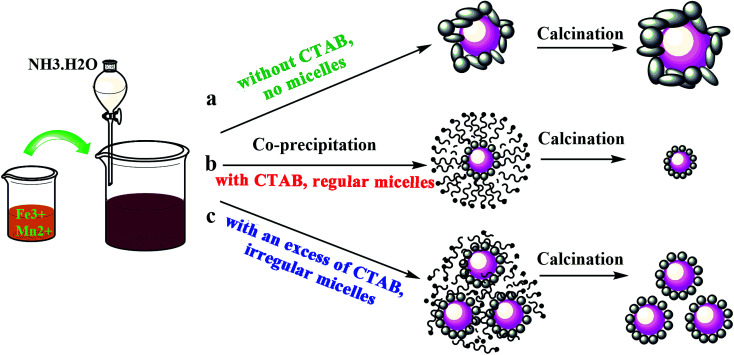
Schematic illustration of the preparation process for the FeMn–CTAB catalysts.

A schematic diagram of the preparation process is shown in Fig. 4. It can be seen that the cationic CTAB surfactant facilely adsorbed on the precipitated particle surfaces through the attraction of opposite charges, leading to the formation of micelle shells, in which the hydrophobic tail group points towards the center nanoparticles and the hydrophilic head group points towards the aqueous solution.^37,38^ The formed micelle shells protect the particles from agglomerating during the preparation process, and were completely evaporated by calcination, with no residual CTAB found in the catalyst.^34–39^ Regular micelle shells were obtained with a moderate amount of CTAB. However, since the precipitation process was unstable and constantly changed, this led to a disordered distribution of the surfactant. Therefore, the formed micelle shells became irregular when an excess amount of CTAB was introduced, as shown in Fig. 4c, which although was not as effective as the former process, it also prevented the particles from agglomerating. This is the reason why the particle size and BET surface area increased for the FeMn–CTAB-1.0 catalyst. This suggests that the optimum molar ratio of CTAB to Fe in the catalyst preparation is 0.8.

**Fig. 5:**
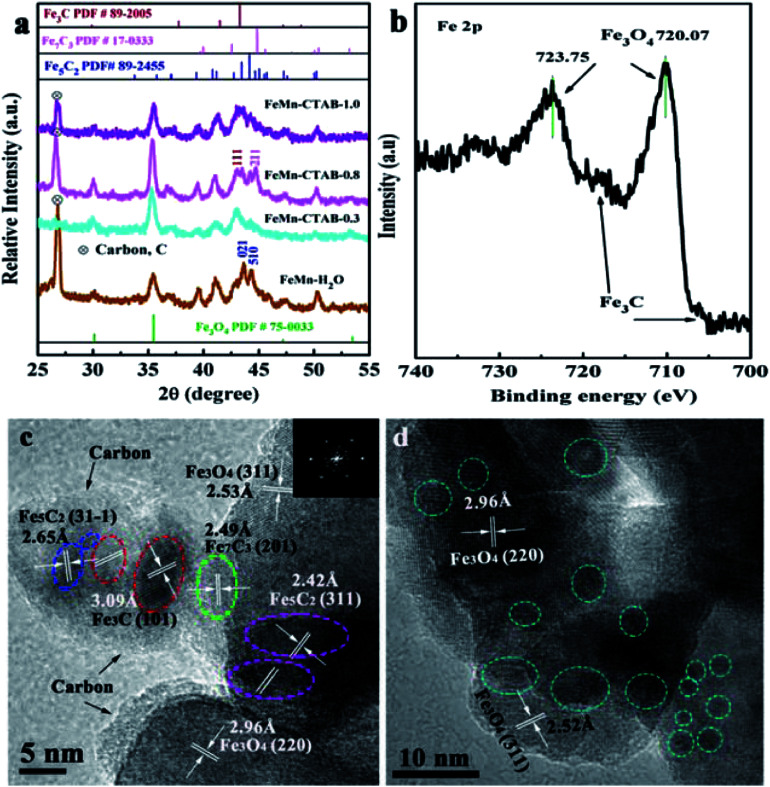
(a) XRD patterns of the spent FeMn catalysts, (b) Fe 2p XPS spectrum of the spent FeMn–CTAB-0.8 sample, and (c and d) (HR)-TEM images of the spent FeMn catalysts.

The structural characterization of the spent FeMn catalysts was carried out after 60 h on stream in the FTO reaction. Fig. 5a shows that the Fe_3_O_4_ and iron carbide species are the dominant phases in the samples. In addition, carbon deposition was observed at a 2*θ* value of 25° (carbon PDF#26-1077), which was due to the Boudouard reaction (2CO → C + CO_2_) in the catalytic reaction process.^40^ The HR-TEM image and Fe 2p XPS spectrum of the spent FeMn–CTAB-0.8 catalyst further proved that the sample mainly consisted of Fe_3_O_4_ and iron carbides, as well as carbon deposits (Fig. 5b and c). The diffraction peaks of the spent catalyst were sharper than those of the as-synthesized α-Fe_2_O_3_ nanoparticles, suggesting that the average size of iron-containing particles increased after the reaction. The TEM results further proved that the average particle size increased from 4.79 to 37.8 nm, as shown in Fig. 2c and S6e.† It is noteworthy that the iron carbides formed during the FTO reaction were present in more than one phase and that this was dependent on the CTAB content. For example, χ-Fe_5_C_2_ (Hägg carbide, PDF#89-2544) was found in the spent FeMn–H_2_O catalyst, while Fe_7_C_3_ (iron carbide, PDF#17-0333) and θ-Fe_3_C (iron carbide, PDF#89-2005) were observed in the spent FeMn–CTAB-0.8 catalyst, which was verified by XRD and HR-TEM, as shown in Fig. 5a and c. This indicates that certain specific surface sites formed when CTAB was employed.

The TEM images in Fig. S6d† show that overlapped particles slightly aggregated, with a size distribution mainly ranging from 20 to 55 nm. The average particle size was 37.8 nm for the spent FeMn–CTAB-0.8, which was larger than that of the other spent samples, as can be seen in Fig. S6,† while the spent FeMn–CTAB-1.0 had the smallest mean size of 24.4 nm among all of the spent samples. It can be considered that small particles tended to aggregate, while the narrow particle-size distribution, with an appropriate particle size of about 9.2 nm, for FeMn–CTAB-1.0 resulted in a decrease in the extent of the particle aggregation. The HR-TEM images of FeMn–CTAB-0.8 exhibit the specific (220) and (311) planes of Fe_3_O_4_, the (31−1) plane of χ-Fe_5_C_2_, the (101) plane of θ-Fe_3_C, and the (202) plane of Fe_7_C_3_. Furthermore, Fig. 5c and d show that the presence of iron carbides located on the surface of Fe_3_O_4_ with a size of 4.3–9.4 nm, which would be very likely to form Fe_3_O_4_@iron carbide core–shell structure nanoparticles.

The Fe 2p XPS spectra of the spent FeMn–CTAB-0.8 catalyst shows two sets of peaks from the Fe_3_O_4_ and χ-Fe_5_C_2_ phases in the range of the Fe 2p_3/2_ (710.10 eV) and Fe 2p_1/2_ (723.75 eV) orbital signals (Fig. 5b), suggesting the reduction and carbonization of α-Fe_2_O_3_,^32^ which is in good agreement with the XRD (Fig. 5a) results. Fig. S7b† shows that the O 1s peak appears at a binding energy of 529.08 eV, and can be deconvoluted into MnO and Fe_3_O_4_, indicating the coexistence of iron–manganese oxide and carbide phases on the surface. Fig. S7d† shows the formation of θ-Fe_3_C after the reaction.

Table 1 summarizes the catalytic activities and hydrocarbon distribution of the FeMn–H_2_O and FeMn–CTAB-*γ* catalysts (*γ* = 0.15/0.3/0.6/0.8/1.0) under the industrially relevant conditions of 320 °C, 1.0 MPa, GHSV = 4200 h^−1^, and a H_2_/CO (v/v) ratio of 1.5, at a TOS = 20 h. As presented in Table 1, the use of the FeMn–H_2_O catalyst resulted in 91.31% CO conversion and 15.29% CH_4_ selectivity, as well as 30.16% C_2–4_^=^ selectivity, of which the ratio of olefin to paraffin (denoted as O/P) in the C_2_–C_4_ range hydrocarbons was as low as 2.19, and the FTY was 1.29 × 10^−5^ mol_CO_ g_Fe_^−1^ s^−1^. For the FeMn–CTAB catalysts, the CO conversion and FTY gradually decreased upon the addition of CTAB, even though there was an increase in the BET surface area due to many of the Fe activity sites being covered by Mn promoter (Fig. 3a). The same results were obtained upon adding the amounts of Mn promoter shown in Table S5† (the molar ratio of CTAB/Fe is 0.8). While the C_2–4_^=^ selectivity and O/P value were found to gradually increase upon the addition of CTAB. When the addition of CTAB was 0.8 (CTAB/Fe, mol mol^−1^), the C_2–4_^=^ selectivity was at its highest at 55.45%, with an O/P value of as high as 7.75. At the same time, the selectivity for C_5+_ hydrocarbons and all oxygenate products (from the 160 °C hot trap and −1 °C cooling trap) significantly decreased.

Combining the above characterization results, it is considered that some specific active sites formed on the surface of the FeMn–CTAB catalysts during the preparation process,^14–16,19^ and resulted in the formation of specific iron carbides, such as χ-Fe_2_C_5_, θ-Fe_3_C, and Fe_7_C_3_ phases, when exposed to the syngas, which might be the main reason for the different catalytic performances observed for the FeMn–CTAB catalysts. In particular, for FeMn–CTAB-0.8, the formation of the θ-Fe_3_C and Fe_7_C_3_ phases was beneficial to the CO dissociation absorption, but suppressed the H_2_ absorption and α-olefin readsorption over the FeMn–CTAB catalysts, contributing to high C_2–4_^=^ selectivity.^19,40^ Many factors may be the cause for the formation of these active phases, especially the specific effects of the Mn promoter.^25^ For example, Mn could affect the electronic state of the surface carbonaceous species, leading to the formation of a special iron carbide phase (θ-Fe_3_C) in the particular catalytic system.^19^ Thus, we prepared a series of FeMn–CTAB catalysts with different loadings of Mn promoter and studied their FTO catalytic performance. The catalyst activity, CO conversion and products distributions are summarized in Table S5.† It can be seen that the C_2–4_^=^ selectivity of FeMn–CTAB-0.8 was at its highest (about 61.21%) when the loading of Mn was 0.1 (Mn/Fe, mol mol^−1^). In addition, the catalyst activity and CO conversion gradually decreased upon an increase in the Mn loading, which could be due to the partial coverage of Mn over the Fe activity sites, which prevents the reactant from making contact with the activity centers (Fig. 3a).^40^

It has been proved that a larger amount of MnO_*x*_ covered the active Fe surface when CTAB was employed, leading to a loss in the number of active Fe sites that are used for CO dissociative adsorption.^19,40,42^ In addition, the active sites were occupied by the absorbed CO because of strong surface basicity, which decreased the rate of CO dissociation.^14,46^ All of these factors account for the decrease in the CO conversion and catalytic activity over the FeMn–CTAB catalysts. However, the addition of an excess of CTAB brought about an increase in the CO conversion and catalyst activity (CTAB/Fe = 1.0, mol mol^−1^), but a distinct decrease in the C_2–4_^=^ selectivity and O/P value, as observed in Table 1, which might be due to the higher exposure of the Fe active phase, as well as the presence of specific iron carbides that formed during the FTO reaction (such as χ-Fe_2_C_5_).^24,41^

**Fig. 6:**
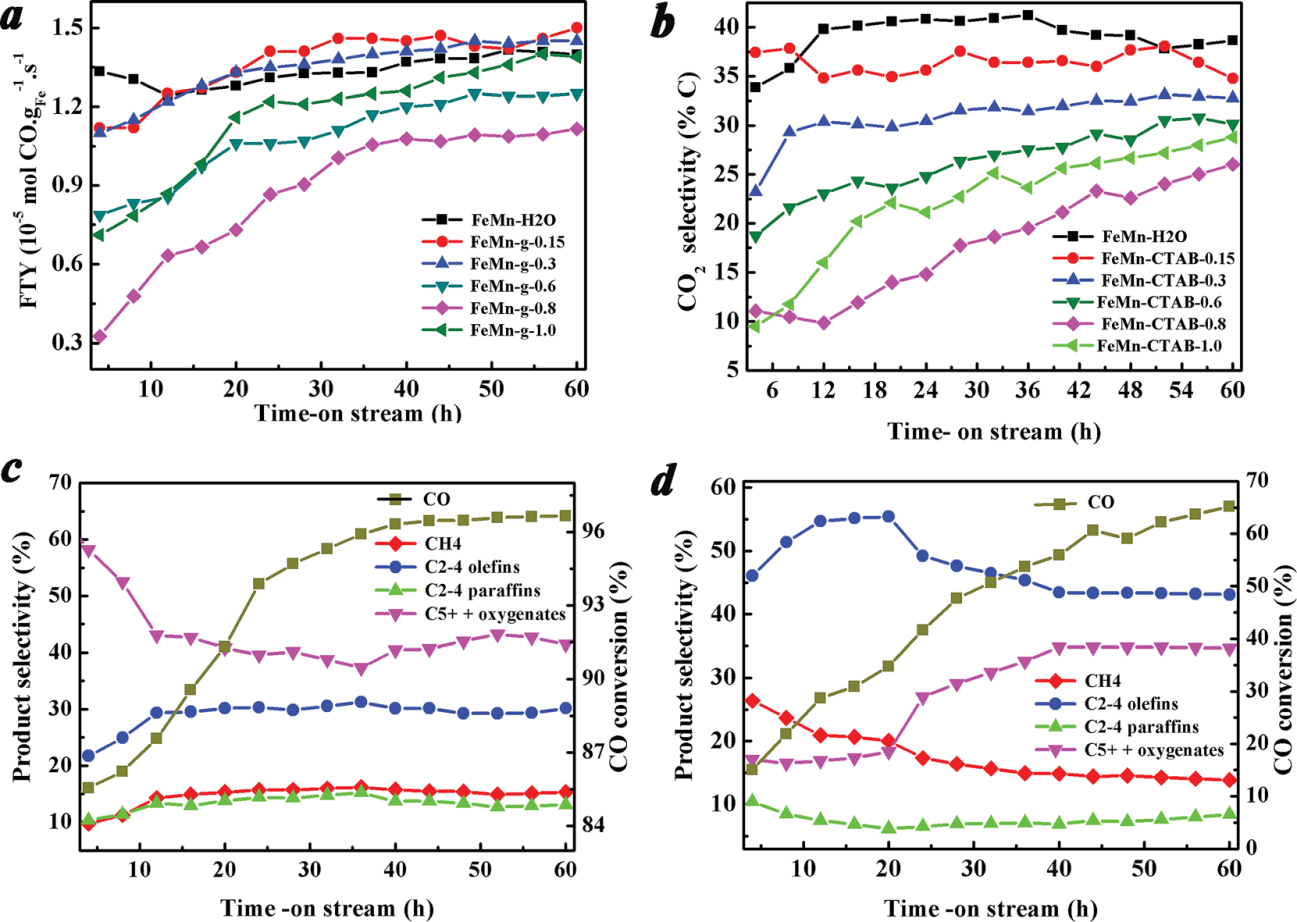
Catalytic performance of the FeMn–H_2_O and FeMn–CTAB-*γ* (*γ* = 0.15/0.3/0.6/0.8/1.0) catalysts as a function of time: (a) catalytic activity, (b) CO_2_ selectivity, and product selectivities of (c) FeMn–H_2_O, and (d) FeMn–CTAB-0.8. Reaction conditions: 320 °C, 1.0 MPa, H_2_/CO = 1.5 (v/v), GHSV = 4200 h^−1^ and TOS = 60 h.

Fig. 6a–d show the catalytic performance during the reaction. It was found that the catalytic activity and the product selectivity changed alongside the on-stream time. Fig. 6a shows that the catalytic activity of all of the calcined catalysts was low at the beginning of the reaction, and increased rapidly in the initial period, then tended to stabilize at a high level. Fig. 6c and d show the CO conversion and the hydrocarbon product selectivity of the FeMn–H_2_O and FeMn–CTAB-0.8 catalysts as a function of the on-stream time. In contrast, the favorable formation of C_2–4_^=^ was observed when CTAB was employed. For the FeMn–CTAB-0.8 catalyst, the C_2–4_^=^ selectivity rose to a maximum (55.45%) and slightly decreased when the reaction proceeded over a longer time, and was then maintained at around 43.5%, which was higher than that of the FeMn–H_2_O catalyst (around 30%). Meanwhile, the formation of C_5+_ hydrocarbons and oxygenate products increased continuously over the first 40 h and seemed to stabilize at 34.8%, which was lower than the value observed for the FeMn–H_2_O catalyst (around 43.5%). The CH_4_ selectivity, as well as the formation of C_2–4_ paraffins, was constant low for both of the catalysts.

Before the reaction, the fresh catalysts were reduced in H_2_ at 400 °C under 1.0 MPa for 12 h, and a large amount of metallic iron formed on the initial catalyst surface, as observed from the H_2_-TPR and *in situ* XRD measurements (Fig. 1c and S3†). On the other hand, the characterization revealed that various carbides species formed on the catalyst surfaces when exposed to syngas, which might be induced by the specific surface active sites of the FeMn catalysts.^14–16,19,40,42^ It has been reported that metallic iron and iron carbides display distinct CO dissociation barriers and binding strengths for C and O atoms,^14,19^ leading to different CO conversions and catalytic activities between the initial reaction stages and after the reaction has proceeded for a longer time (Fig. 6a–d).^14,32,33,40,42^ The same applies to the product selectivity, which is influenced not only by the initial state of the catalyst surface, but also longer reaction times.^14–16^ These results further demonstrated that the changed iron catalyst surface, including the growth of iron carbide particles, carbide structure, carbon accumulation (Fig. 5a–c), and binding strength between some atoms, had a great influence on the catalytic performance.^14–16,40^

The product distributions of FeMn–H_2_O were well fitted using the traditional Anderson–Schulz–Flory (ASF) model, while great deviation from the traditional ASF model was found for FeMn–CTAB-0.8, as shown in Fig. S9,† due to the positive effects of CTAB on the catalyst structure, such as small particle size, and a large amount of exposed Mn.

## 4. Conclusions

CTAB was found to have a significant influence on the catalytic performance of a Mn-promoted iron FTO catalyst. Characterization revealed that the surfactant had a great effect on the catalyst structure, composition and chemical state, leading to the formation of various iron carbide species on the surface-specific activity sites when the catalysts were exposed to syngas. Iron carbides served as the active phase, which was essential for enhancing the catalytic activity and the selectivity for light olefins. In general, this work details a simple method for the design of a highly selective FTO catalyst under industrially relevant conditions (320 °C, 1.0 MPa, H_2_/CO ratio of 1.5 (v/v), GHSV = 4200 h^−1^). In addition, it further confirms the role of specific iron carbides in the formation of light olefins and also helps to understand the relationship between the catalyst structure and its catalytic performance. Moreover, the CTAB surfactant-assisted Fe catalyst can be used for the high selectivity of light olefins in the industrially appealing FTO process.

## Conflicts of interest

There are no conflicts to declare.

## Supplementary Material

RA-008-C8RA04622K-s001
