# Supplementary material for: Enhancing the light olefin selectivity of an iron-based Fischer–Tropsch synthesis catalyst by modification with CTAB
Source: RSC Adv. 2018 Sep 14;8(56):32073–83. doi: 10.1039/c8ra04622k (PMC9085730; doi:10.1039/c8ra04622k)
Supplement: RA-008-C8RA04622K-s001 [file RA-008-C8RA04622K-s001.pdf]

Electronic Supplementary Material (ESI) for RSC Advances.
This journal is © The Royal Society of Chemistry 2018

Supporting Information

Enhancing Light Olefins Selectivity of Iron-Based Fischer-Tropsch Synthesis Catalyst by the Modulation of CTAB

Chuanxue Zhu [a], Yingxin Liu [b], Chao Huo [a,*], Huazhang Liu [a]

[a] Institute of Industrial Catalysis, Zhejiang University of Technology, Hangzhou 310032, Zhejiang, PR China

[b] Research and Development Base of Catalytic Hydrogenation, College of Pharmaceutical Science, Zhejiang University of Technology, Hang-zhou 310032, Zhejiang, PR China

*Corresponding Author: Tel.: +86-571-88320815. Fax: +86-571-88320815,E-mail: chaohc@zjut.edu.cn

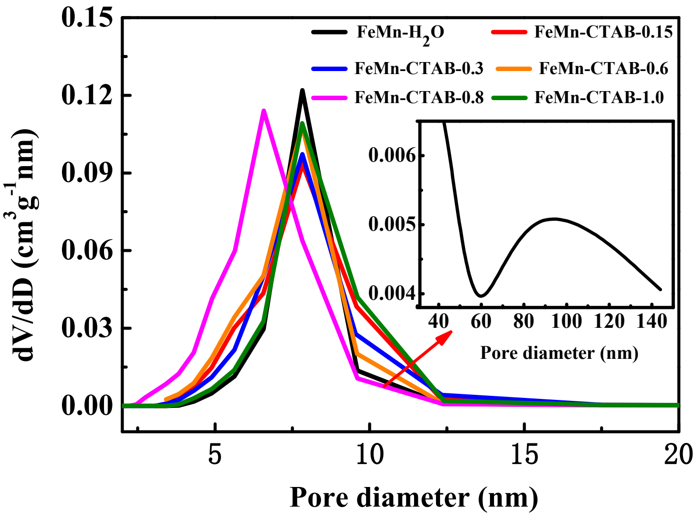

Fig. S1. Pore size distribution of the calcined FeMn catalyst with different addition of CTAB.

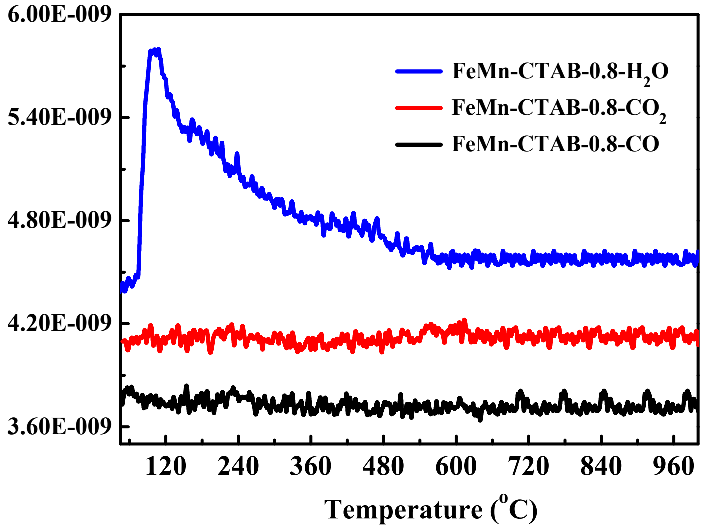

Fig. S2. TG-MS profiles of the calcined FeMn-CTAB-0.8 catalyst in air.

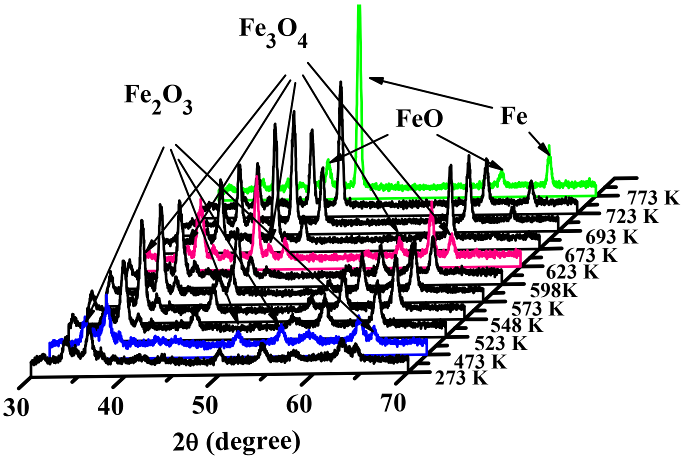

Fig. S3. *In-Situ* XRD patterns of the calcined FeMn-CTAB-0.8 catalyst.

Fig. S3 displays the *in-Situ* XRD patterns of FeMn-CTAB-0.8 recorded from 25 °C to 550 °C. It revealed that the reduction process of α-Fe_2O_3 to Fe_3O_4 started at 275 °C and almost completed at 400 °C. The characteristic peaks intensity of Fe_3O_4 phase at 2θ=53.4, 56.9° (PDF#88-0315) gradually decreased from 300 °C to 425 °C as observed. At the same time, the peaks of FeO phase at 2θ=41.921, 60.74° (PDF#06-0615) had emerged at 400 °C, indicating that the Fe_3O_4 phase was transformed into FeO. Moreover, the metallic Fe phase can be found at 450 °C with the growth of its nanoparticles size when temperature was further raised. No crystal phase detected for the manganese oxides phase.

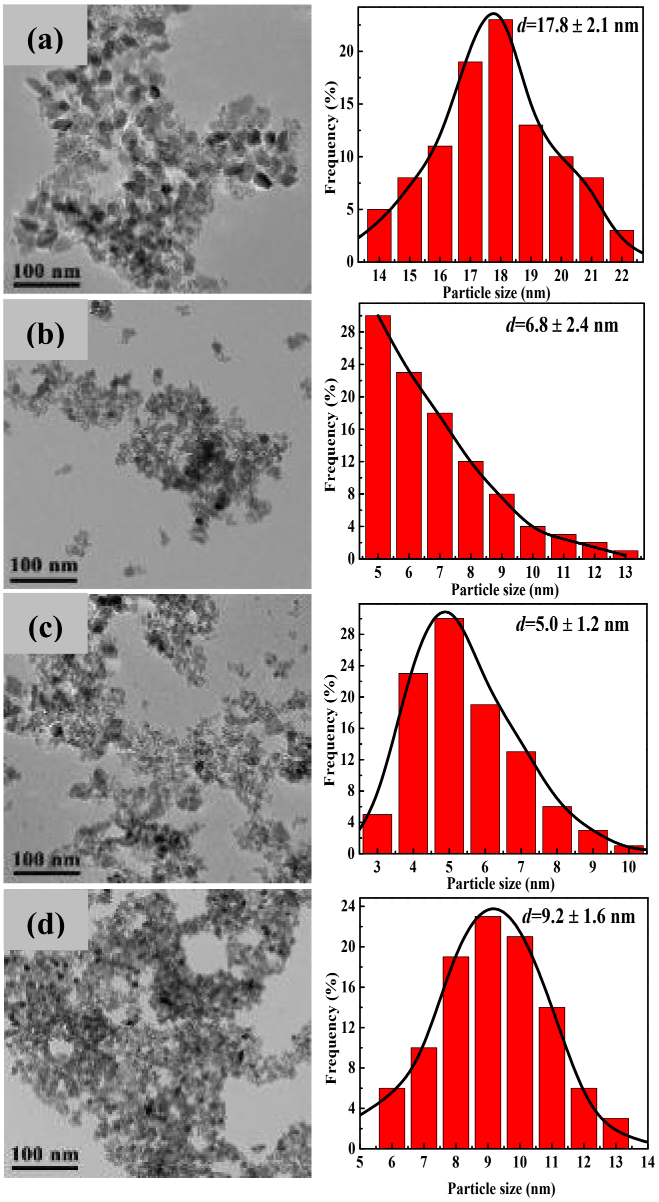

Fig. S4.TEM images and particle size distribution of the calcined FeMn catalysts: (a) FeMn-CTAB-0.15, (b) FeMn-CTAB-0.3, (c) FeMn-CTAB-0.6, (d) FeMn-CTAB-1.0.

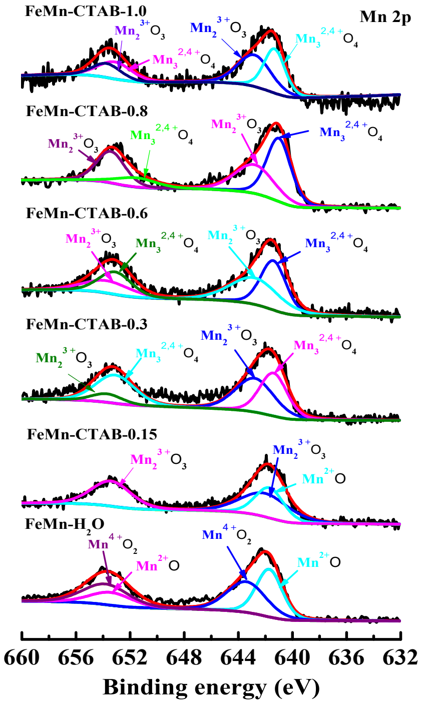

Fig. S5. The elaboration of Mn 2p results of the FeMn catalysts as-prepared.

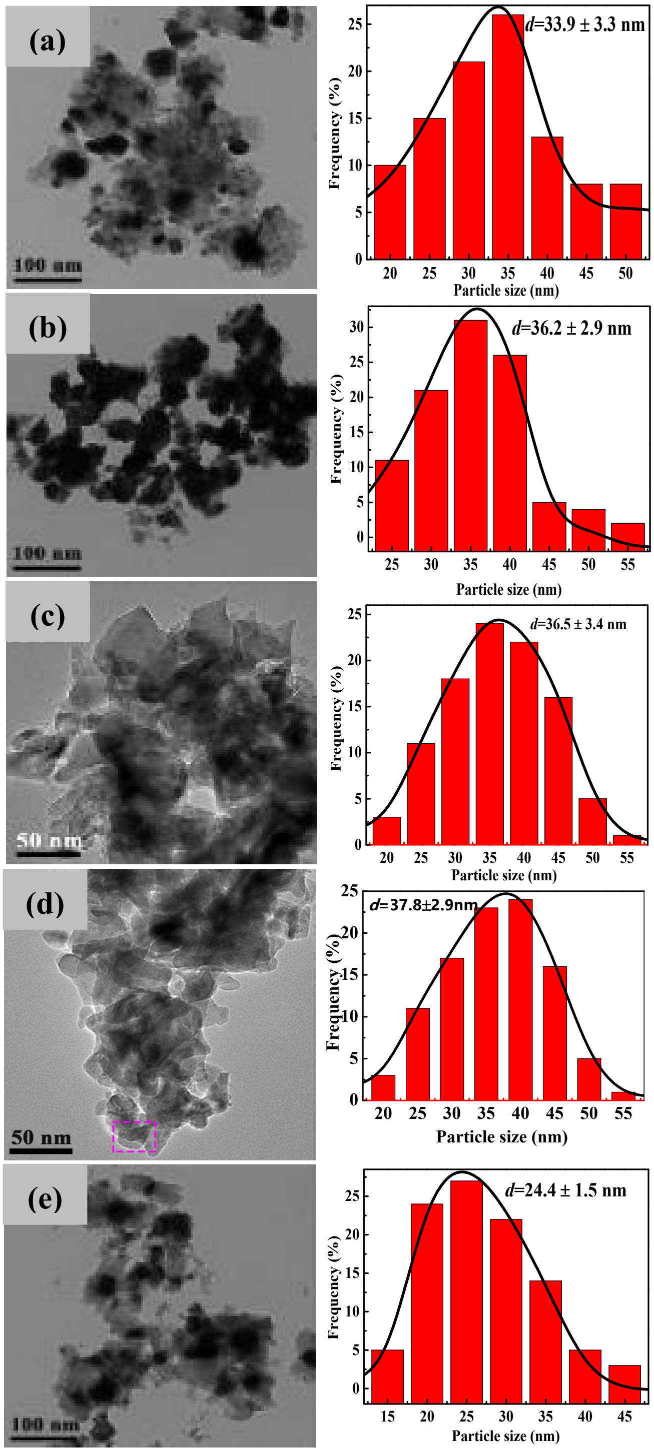

Fig. S6.TEM images and particle size distribution of the spent FeMn catalysts: (a) FeMn-0, (b) FeMn-CTAB-0.3, (c) FeMn-CTAB-0.6, (d) FeMn-CTAB-0.8 (e) FeMn-CTAB-1.0.

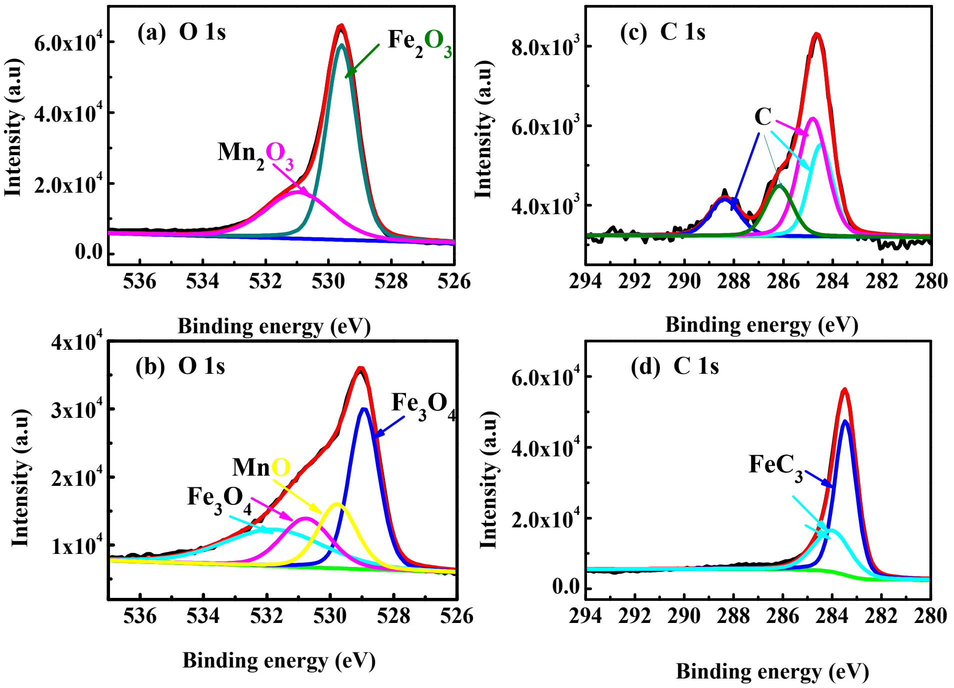

Fig. S7. The elaboration of the FeMn-CTAB-0.8: O 1s (a) Calcined sample, (b) Spent sample; C 1s (c) Calcined sample, (d) Spent sample.

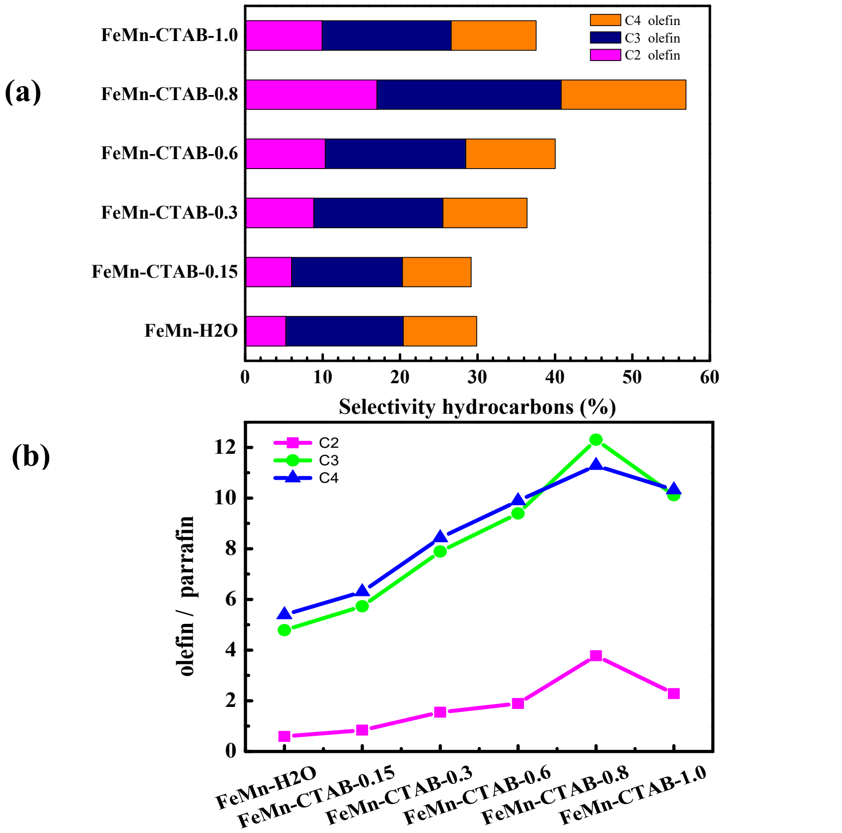

Fig. S8. (a) C_2-C_4olefins s selectivity at TOS=60 h, (b) Ration of olefin to paraffin in C_2-C_4hydrocarbons as a function of time.

Light olefins may have a very low tendency for secondary reactions except for ethylene.[1,2] As the Fig. S7a shown, the selectivity of $C_2^=$ of 5.62 % (molar fraction) less than that of $C_3^=$ (14.99 %) and $C_4^=$ (9.61 %) for FeMn-H_2O, which might be caused by more ethenes than the other alkenes are reabsorbed onto the active site to initiate a new chain growth reaction. Similar to the FeMn-CTAB catalysts. On the other hand,Fig. S8b shows ethenes exhibited more prone to hydrogenation than propylenes and butylenes, the O/P value arrived to maximum for FeMn-CTAB-0.8, suggesting the suppression of the H_2 absorption and olefin readsorption. As a result, more light olefins were produced for the Mn-promoted Fe catalyst.

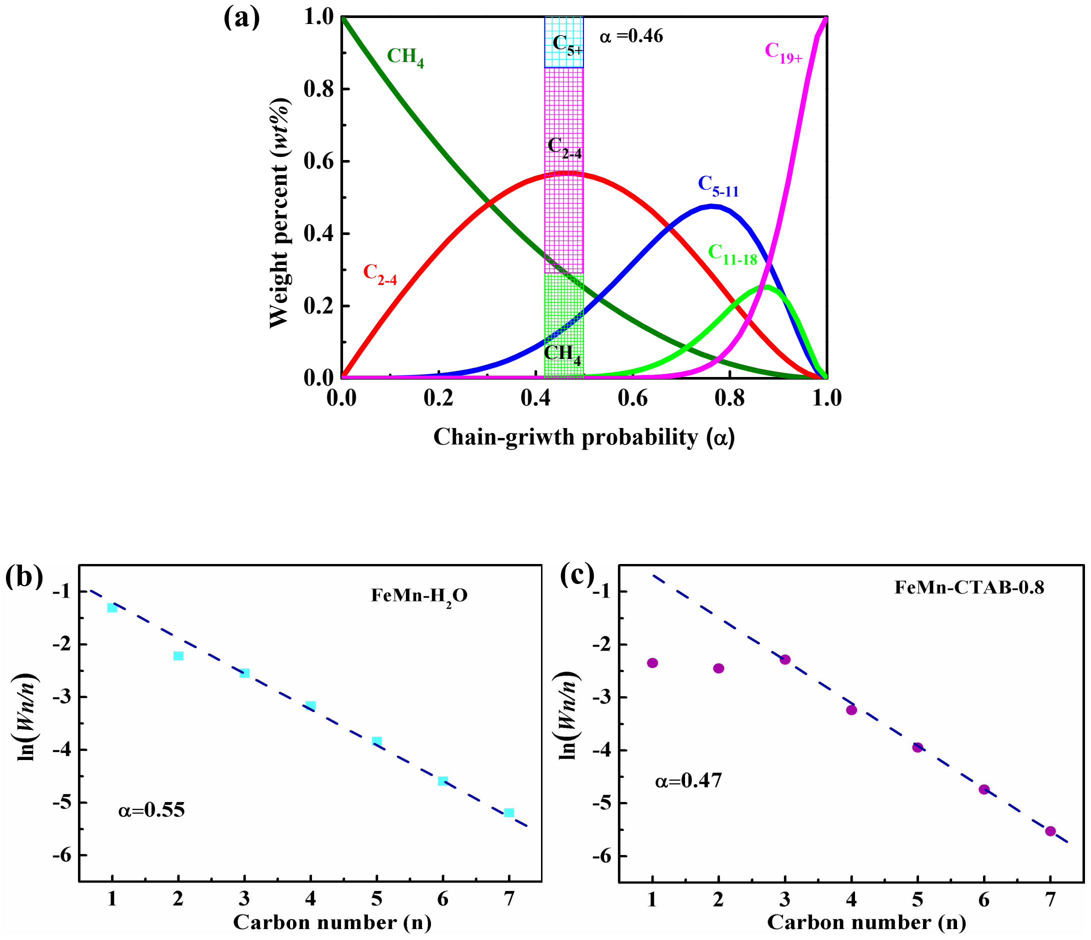

Fig. S9. (a) Product distribution according to the Anderson–Schulz–Flory (ASF) model, (b)-(c) Typical product plots (ln(Wn/n) versus n) obtained using our FeMn catalyst synthesized with different amount of CTAB .Catalysts were *in-situ* reduced at 400 °C for 12 h and tested at 320 °C, 1.0 MPa, GHSV=4200 h^{-1}, H_2/CO=1.5(*v/v*).

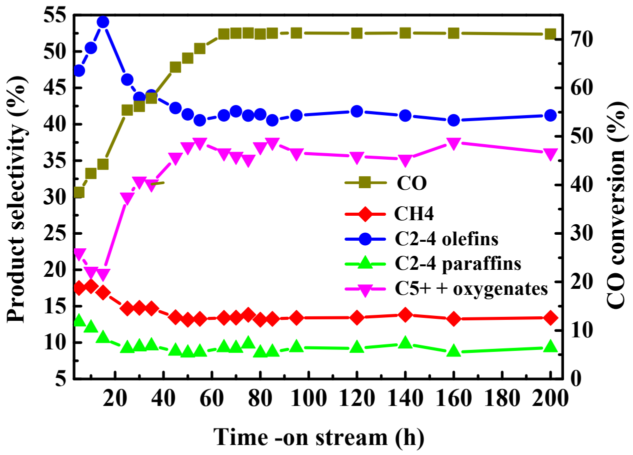

Fig. S10. Evaluation of the long-term stability of FeMn-CTAB catalyst.

Table S1. Elemental analysis, surface areas, pore parameters and particles size for FeMn catalysts with different addition of CTAB.

Catalysts	XRF	EDS	XPS	BET Surface area	Pore Size	Pore Volume	Crystallite Size[a]
	Atom %			(m^2/g)	(nm)	(cm^3/g)	(nm)
FeMn-H_2O	0.103	0.104	0.139	73.220	7.828	0.235	18.1
FeMn-CTAB-0.15	0.091	0.088	0.147	105.490	7.820	0.260	12.3
FeMn-CTAB-0.3	0.091	0.092	0.170	123.567	7.833	0.300	7.2
FeMn-CTAB-0.6	0.092	0.091	0.129	127.368	7.816	0.293	7.1
FeMn-CTAB-0.8	0.081	0.079	0.119	135.652	6.573	0.303	6.1
FeMn-CTAB-1.0	0.090	0.088	0.152	118.080	7.815	0.298	9.1

[a] Calculated from the Debye–Scherrer equation for hematite Fe_2O_3.

Table S2. Peak areas of manganese and iron oxides for FeMn catalysts in H_2-TPR profiles.

Catalysts	T (℃)	Peak Area	T (℃)	Peak Area	T (℃)	Peak Area	T (℃)	Peak Area
FeMn-H_2O	N.A	N.A	366	38	514	28	576	13
FeMn-CTAB-0.15	326	20	359	17	510	36	613	36
FeMn-CTAB-0.3	330	27	370	14	N.A	N.A	609	56
FeMn-CTAB-0.6	330	25	363	13	N.A	N.A	598	52
FeMn-CTAB-0.8	326	35	355	10	N.A	N.A	595	57
FeMn-CTAB-1.0	328	29	360	14	N.A	N.A	597	58

Table S3. Peak areas of medium strong base and strong base for FeMn catalysts.

Catalysts	Temperature (℃)	Peak Area	Temperature (℃)	Peak Area
FeMn-H_2O	277	60	452	139
FeMn-CTAB-0.15	267	95	471	216
FeMn-CTAB-0.3	259	99	476	246
FeMn-CTAB-0.6	254	110	483	309
FeMn-CTAB-0.8	244	183	489	440
FeMn-CTAB-0.10	227	262	493	517

Table S4. XPS binding energies and concentrations for FeMn catalysts.

Catalysts	Binding energies(eV) and concentration (in atomic %)					
	Fe 2p 3/2	(%)	Mn 2p 3/2	(%)	O1s	(%)
FeMn-H_2O	710.80	27.21	642.10	3.79	529.90	53.12
FeMn-CTAB-0.15	710.42	25.28	641.73	3.72	529.74	47.58
FeMn-CTAB-0.3	710.53	24.80	641.70	4.21	529.74	47.75
FeMn-CTAB-0.6	710.60	27.36	641.60	3.52	529.78	49.22
FeMn-CTAB-0.8	710.54	29.04	641.28	3.47	529.60	50.78
FeMn-CTAB-1.0	710.7	26.97	641.75	4.11	529.70	48.30

Table S5. Catalytic performance of the FeMn catalyst with different ratio Mn/Fe [a].

Catalysts	FeMn-0	FeMn-0.06	FeMn-0.10	FeMn-0.15	FeMn-0.20
CO conversion (C %)	86.63	30.92	24.51	28.41	42.36
FTY [b] (10^{-6} $mol_{co}\cdot g_{Fe}^{-1}\cdot s^{-1}$)	9.95	6.65	5.02	5.11	5.24
CO_2 selectivity (C %)	38.61	11.96	17.94	19.34	27.19
Products selectivity (% C, CO_2-free)					
CH_4	13.31	20.66	21.25	24.0	16.74
C_2	16.58	22.62	22.09	19.91	20.30
C_3	21.60	25.96	27.02	28.37	24.94
C_4	13.77	16.44	19.23	21.44	16.99
C_{2-4} olefins	39.80	58.16	61.21	53.79	49.99
C_{2-4} paraffins	12.14	6.85	7.13	15.92	12.20
C_{5+} + oxygenates [d]	34.74	14.32	10.41	6.29	21.03
olefin/paraffin ratio					
C_2	1.21	5.38	4.99	1.06	1.71
C_3	6.33	12.10	13.03	6.89	7.71
C_4	7.14	11.43	11.68	7.08	8.05
C_{2-4}	3.28	8.49	8.59	3.38	4.08

[a] Catalysts were *in-situ* reduced at 400 °C for 12 h and tested at 320 °C, 1.0 MPa, GHSV=4200 h^{-1}, H_2/CO=1.5(*v/v*); CTAB/Fe=0.8 (mol/mol) in the preparation.

[b] FTY (Iron time yield) mol of CO converted to hydrocarbons (excluding CO_2) per time (s) per weight of iron (g).

[d] C_{5+} is hydrocarbon which was analyzed online by gas chromatograph (GC); Oxygenates is from 160 °C hot trap and -1 °C cooling trap.

Table S6. Catalytic performance of the FeMn catalyst with different GHSV [a].

GHSV h^{-1}	CO conversion (C mol %)	CO_2 selectivity (C mol %)	hydrocarbon selectivity (C mol %,CO_2-free)				O/P [c]
			CH_4	C_{2-4} olefins	C_{2-4} paraffins	C_{5+}+ Oxygenates [d]	
3600	47.25	22.59	18.32	56.58	8.72	16.38	6.49
4200	16.95	10.63	21.52	61.99	6.75	9.74	9.18
4800	18.97	7.49	21.39	55.65	6.48	16.48	8.59
6000	28.21	9.65	20.39	50.40	6.39	22.82	7.89

[a] Catalysts were *in-situ* reduced at 400 °C for 12 h and tested at 320 °C,1.0 MPa, $H_2/CO=1.5(v/v)$, at initial reaction stages.

[b] CTAB/Fe=0.8, Mn/Fe=0.10 (mol/mol).

[c] O/P represents the molar ratio of olefin to paraffin in the C_2-C_4 range hydrocarbons.

[d] C_{5+} is hydrocarbon which was analyzed online by gas chromatograph (GC); Oxygenates is from 160 ℃ hot trap and -1 ℃ cooling trap.

References

[1] X. Zhang, Y. Liu, G.-Q. Liu, K. Tao, Q. Jin, F.-Z. Meng, D. Wang, N. Tsubaki, *Fuel* **2012**, *92*, 122-129.

[2] J.-B. Li, H.-F. Ma, H.-T. Zhang, Q.-W. Sun, W.-Y. Ying, D.-Y. Fang, *Acta Phys.-Chim. Sin*. **2014**, *30*, 1932-1940
